# Dynamics of Scroll Wave in a Three-Dimensional System with Changing Gradient

**DOI:** 10.1371/journal.pone.0152175

**Published:** 2016-03-31

**Authors:** Xiao-Ping Yuan, Jiang-Xing Chen, Ye-Hua Zhao, Gui-Quan Liu, He-Ping Ying

**Affiliations:** 1 Information Engineering School, Hangzhou Dianzi University, Hangzhou 310018, P.R. China; 2 Department of Physics, Hangzhou Dianzi University, Hangzhou 310018, P. R. China; 3 Department of Physics, China Jiliang University, Hangzhou 310018, P. R. China; 4 Department of Physics, Zhejiang University, Hangzhou 310027, P. R. China; Lanzhou University of Technology, CHINA

## Abstract

The dynamics of a scroll wave in an excitable medium with gradient excitability is studied in detail. Three parameter regimes can be distinguished by the degree of gradient. For a small gradient, the system reaches a simple rotating synchronization. In this regime, the rigid rotating velocity of spiral waves is maximal in the layers with the highest filament twist. As the excitability gradient increases, the scroll wave evolutes into a meandering synchronous state. This transition is accompanied by a variation in twisting rate. Filament twisting may prevent the breakup of spiral waves in the bottom layers with a low excitability with which a spiral breaks in a 2D medium. When the gradient is large enough, the twisted filament breaks up, which results in a semi-turbulent state where the lower part is turbulent while the upper part contains a scroll wave with a low twisting filament.

## Introduction

Rotating spiral waves in 2D and scroll waves in 3D are observed in many physical, chemical, and biological dissipative systems. Spatiotemporal pattern formation, pattern stability, and corresponding control strategies are among the major topics in the study of distributed active systems [1–3], convective systems [4, 5], and fibrillation in cardiac tissue, which is closely related to the onset of the scroll wave turbulence [6–9]. Scroll waves or regular scroll ring patterns have been obtained in excitable media such as the FitzHugh-Nagumo model and the Belousov-Zhabotinsky reaction [10]. However, such patterns are unstable and eventually disappear. Another novel 3D oscillating pattern developing around a central core and periodically returning to the original site of incipience is being recreated regularly [11]. These patterns were proposed as the main cause of turbulence in the heart, leading to ventricular fibrillation and sudden cardiac death [12, 13].

The interest in the dynamics of these rotating scroll waves or scroll-wave turbulence in 3D cardiac tissue has significantly broadened over the years as developments in experimental or numerical techniques have permitted them to be observed in much detail [14, 15]. Scroll waves are 3D excitation vortices rotating around 1D phase singularities called filaments. Filament dynamics describes ventricular tachycardia and, in particular, ventricular fibrillation [16]. Filaments are dynamic objects that move with local speeds in proportion to the local curvature. Previous studies have investigated the pinning of closed filament loops to inert cylindrical heterogeneities in a chemical reaction-diffusion system in 3D regimes [17, 18]. These studies showed that the filament wraps itself around the heterogeneity and thus avoids contraction and annihilation. Furthermore, parameters that can mimic the inhomogeneity of the heart can be tuned such parameters include period force, simple noise, and gradient. External gradients that are oriented parallel to the filament can drive the scroll wave to drift and twist [19–21], control the spatial orientation and lifetime of scroll rings [22, 23] and simulate the instability of the scroll wave. Experimentally, scroll waves have been exposed to gradients of chemical concentration, temperature and electrial fields [9, 19, 21, 23, 24].

Although the mechanism of scroll wave instability has been studied in detail, only few studies have analyzed the evolution of scroll wave patterns. In this paper, we examine the effects of spiral wave propagation on the tension of 3D scroll wave filaments in an excitable medium. The pattern dynamics by increasing the gradient of excitability can be well understood by the effect of the phase twist of scroll wave filaments. In our simulation studies, three parameter regimes can be distinguished by the degree of gradient. Then, a reasonable explanation to these phenomena is given.

## Results

The simulation of an excitable medium is performed in terms of a modified FitzHugh-Nagumo model (the Bär model)[25]. The two variable reaction-diffusion model is given by

$$\begin{array}{ll} \frac{\partial u}{\partial t} & =\;f(u,v)+\nabla^{2}u\\ \frac{\partial v}{\partial t} & =\;g(u,v). \end{array}$$ (1)

The 3D Laplace operator can be expressed as ∇^2^ = ∂^2^/∂*x*^2^ + ∂^2^/∂*y*^2^ + ∂^2^/∂*z*^2^, and the variables *u* and *v* can be viewed as the “fast” and “slow” variables respectively. In this model, $f\left(u,v\right)=\frac{1}{\varepsilon }u\left(1-u\right)\left[u-\left(v+b\right)/a\right]$, and *g*(*u*, *v*) describes a delayed production of inhibitor with *g*(*u*, *v*) = −*v* for 0 ≤ *u* < 1/3, *g*(*u*, *v*) = 1 − 6.75*u*(*u* − 1)^2^ − *v* for 1/3 ≤ *u* < 1, and *g*(*u*, *v*) = 1 − *v*, for *u* ≥ 1. Numerical simulations are conducted on 256 × 256 × 32 3D grid points employing the explicit Euler method. The space and time steps are △*x* = △*y* = △*z* = 1/256 and △*t* = 0.02, respectively. No-flux condition is imposed on the boundaries. Parameters a = 0.84 and b = 0.07 are fixed to ensure the medium is excitable. *ε* is the ratio of their temporal scales, which characterizes the excitability of the medium. The medium with gradient excitability is constructed by continuously increasing the parameter *ε* of every layer (32 layers). The parameter *ε*_1_ = 0.04 is fixed, whereas *ε*_32_ is changed in the simulation to tune the gradient excitability. Obviously, the *ε* in the i-th layer can be calculated from *ε*_*i*_ = 0.04 + (*i* − 1) × (*ε*_32_ − 0.04)/31.

**Fig 1:**
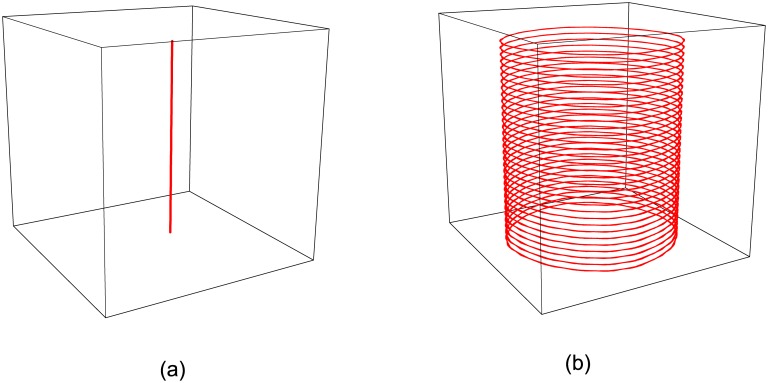
(Color online) (a) The tip filament of a scroll wave at the moment *t* = 200; (b) The circular trajectory of the spiral tip in each layer. Excitability parameter is *ε* = 0.04.

The scroll wave is formed by spiral waves from each layer. Thus, showing the dynamics of a spiral wave when *ε* is changed is necessary. The medium supports steady rotating spiral waves in the range 0.01 < *ε* < 0.06. The spiral waves undergo a transition from steady rotation to meandering at 0.06 < *ε* < 0.07. In the range *ε* > 0.07, spiral waves break up and the system quickly falls into a turbulent state. In our simulation, a scroll wave in homogeneous medium with *ε* = 0.04 is selected as a given initial state. The tip filament at one moment is a straight line as shown in Fig 1(a), and the trajectories of the spiral tips in each layer are circles with the same radius as shown in Fig 1(b). Therefore, the spiral waves in each layer rotate at an identical frequency. The scroll wave is stable in this case.

**Fig 2:**
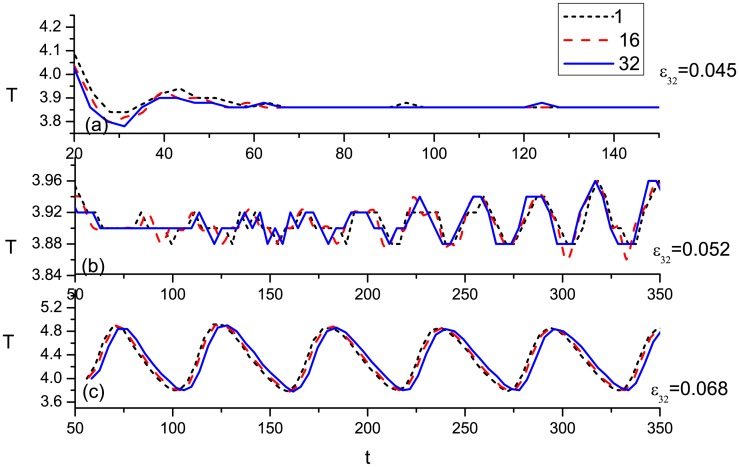
(Color online) The time evolution of the period of spiral waves are plotted from the 1th, 16th, and 32th layer. Three excitability gradient are illustrated in (a) *ε*_32_ = 0.045, (b) *ε*_32_ = 0.052, and (c) *ε*_32_ = 0.068.

The rigid scroll wave becomes unstable if the excitability gradient along the filament exceeds a critical value. The frequency gradient may appear because excitability induces diverse spiral frequencies in different layers. For three values of the gradient parameter *ε*_32_, the time evolution of the periods of spiral waves plotted from three layers is illustrated in Fig 2. When the gradient is small, i.e., *ε*_32_ = 0.045, the system reaches a rigid rotation state after a short time evolution, as shown in Fig 2(a). The interactions between each layer result in a stable synchronization with a period *T* = 3.86. The period of the spiral wave is 3.82 with *ε* = 0.04 in the first layer and 4.0 with *ε* = 0.045 in the 32nd layer. When *ε*_32_ is further increased to 0.052, simulation shows that the system reaches another synchronous state after the evolution time is prolonged. Although the spiral waves in every layer are rigidly rotating with different frequencies, their interactions lead to a meandering behavior in every layer. Different from the first synchronization, this is a meandering synchronous state that can be observed from the evolution of periods in Fig 2(b). The spiral wave in the top layer with the highest excitability is the first to reach the synchronous state. Along the gradient, the time needed to achieve the synchronous state is increased (Fig 2 (b)).

In a 2D system, the critical value of *ε* = 0.06 characterizes the boundary between the rigid rotating spiral and the meandering spiral. As Δ*ε* becomes large enough, e.g., *ε*_32_ = 0.068, meandering spiral waves appear in the bottom layers while rigid rotating spirals initially appear at the top. Fig 2(c) shows that the coupling between layers results in a meandering synchronous state rapidly. Simulation results show that the radius of the tip orbit of spirals in every layer expands.

**Fig 3:**
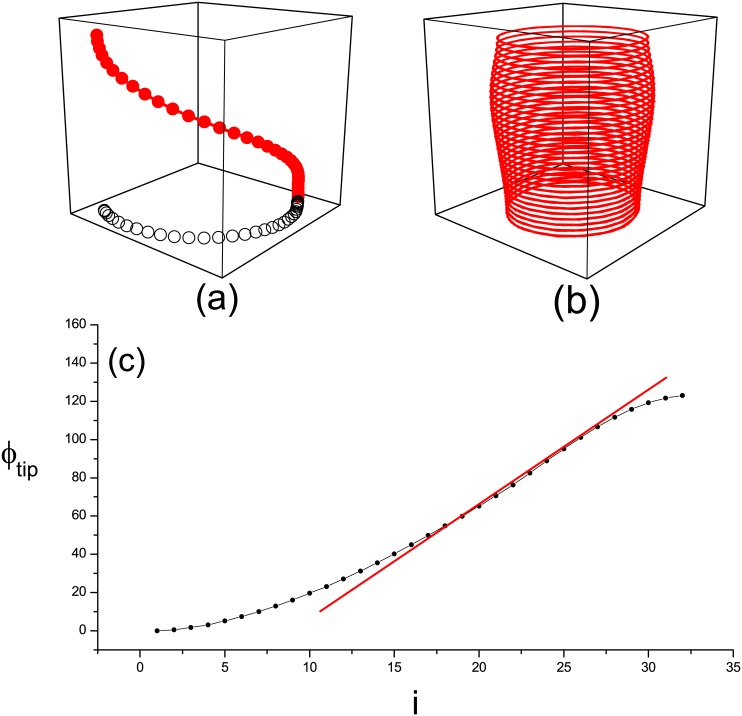
(Color online) (a) Filament of tips in three dimensional space for the moment *t* = 200 at *ε*_32_ = 0.045. The two lines show the same filament from two different viewpoints. The open circle is the one that projection into the (*x*, *y*− plane). (b) The tip trajectory of every layer. (c) The phase filament twist angle of each layer correspond to the top one.

To provide insights into the mechanism underlying the transition of synchronous mode via changing *ε* gradient in the simulation discussed above, we study the dynamics of filament in a 3D system. A filament of the first type of synchronization is shown in Fig 3(a) with *ε*_32_ = 0.045. A twisted filament appears instead of a straight line when the system is synchronous. Obviously, the length of the filament increases. In this case, the tip orbits of spirals in every layer are still circles but with different radii (Fig 3(b)). The velocity of each layer’s spiral tip is proportional to the radius *R* because the system is synchronous with the same spiral period *T* (Fig 2(a)). Therefore, the spiral velocity in the top layer is faster than that in the bottom layer. The maximum velocity appears in the middle layers (Fig 3(b)) because the degree of filament twist in this region is the maximum. This point can be verified in Fig 3(c), where the twist angles *ϕ*_*tip*_ of the spiral tip in each layer with respect to that in the top layer are plotted. The values of *ϕ*_*tip*_ increase with the layer *i* (*ϕ*_*tip*−32_ ≈ 120° at *i* = 32). However, its slope shows a non-monotonous increase. The slope obviously increases first and then decreases. The knee point is clearly in the middle layers.

**Fig 4:**
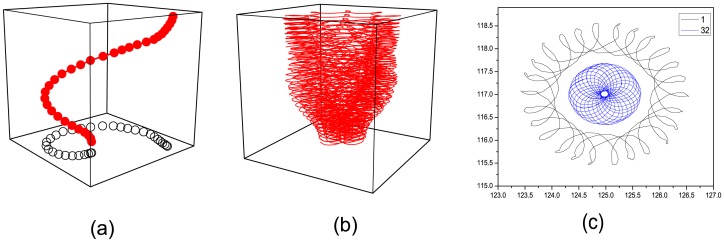
(Color online) (a) Filament of tips in three dimensional space for the moment *t* = 500 at *ε*_32_ = 0.052; (b) The outward meandering tip trajectories of every layer; (c) The trajectories of the top and bottom layer from the outside to the inside.

Compared with that in Fig 3(a), the degree of filament twist is more obvious in Fig 4(a), where *ε*_32_ is increased to 0.052. In this case, the spirals in the top layers can no longer drive those in the lower ones. Consequently, rigid synchronization cannot be maintained. Instead, a meandering synchronization is achieved. The top layer with larger excitability is the first to achieve the meandering state (Fig 2(b)). Then, the rigidly rotating spirals in the lower layers are driven to gradually reach the meandering synchronous state from top to bottom. The delay along the Z-axis reveals that the interaction resulting in synchronization is one by one. Notably, the values of *ε* in all layers are still smaller than 0.06, that is, below the hopf-bifurcation point in the 2D system. Subsequently, the interaction leads to the outward flowerpot-like trajectories of the spiral tip in a counter-clockwise direction (Fig 4(b) and 4(c)). The rotating velocity decreases along the Z-axis, as shown in Fig 4(c).

**Fig 5:**
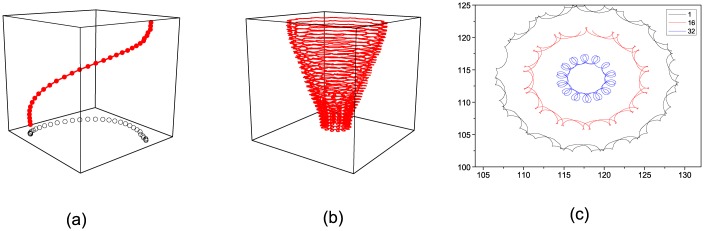
(Color online)(a) Filament of tips in three dimensional space for the moment *t* = 500 at *ε*_32_ = 0.068; (b) The outward meandering tip trajectories of every layer; (c) The trajectories of the first, 16th and the 32th layer along the outward direction respectively.

Then, we increase *ε*_32_ across 0.6 so that two dynamical regimes along the Z-axis co-exist initially. The top layers (*ε* ∈ [0.04, 0.06]) have rigid spirals, and the lower layers (*ε* ∈ [0.06, 0.068]) have meandering spirals. The filament twists quickly (Fig 5(a)) after a very short evolution time. The meandering behavior of the spiral tips in each layer becomes more pronounced, which can be observed from the expanded radii of orbits in Fig 5(c). The trajectories of tips in Fig 5(b) illustrate another obvious phenomenon, that is, the ratio *r*_1_/*r*_32_ increases as *ε*_32_.

**Fig 6:**
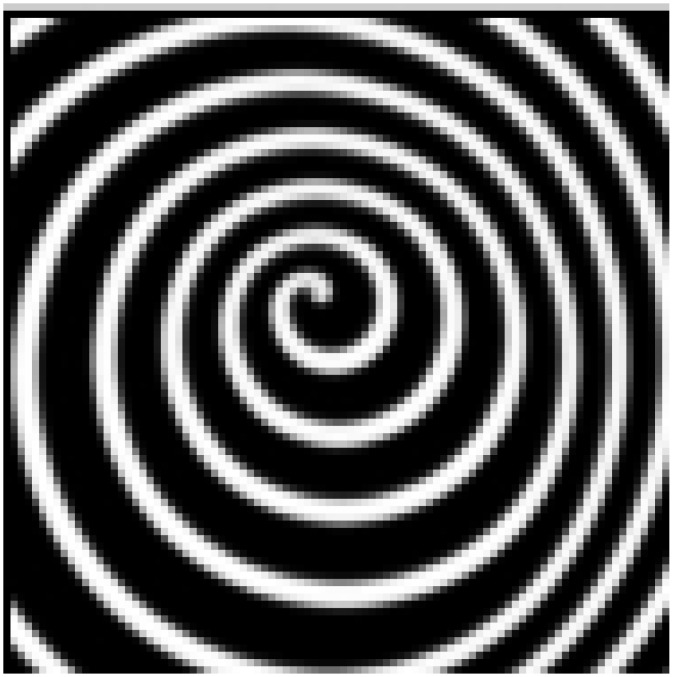
The snapshot of the 32th layer at *t* = 500 with the excitability parameter *ε*_32_ = 0.079.

**Fig 7:**
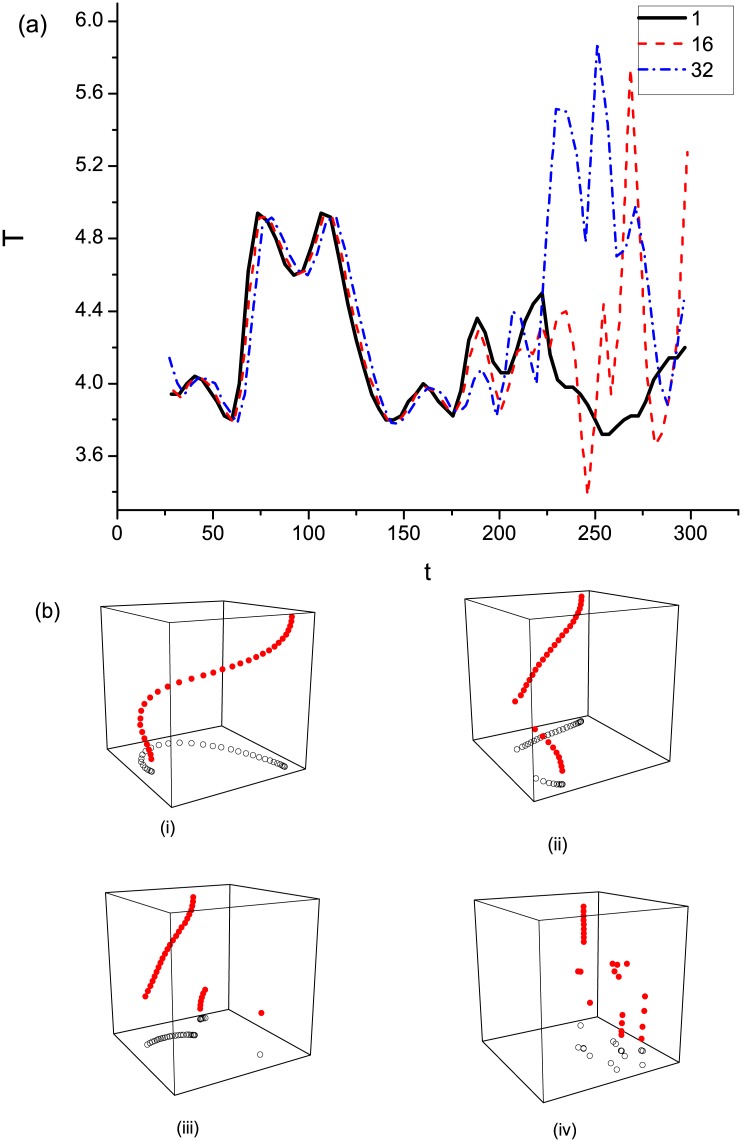
(Color online)(a) The time evolution of the period of three different layers at *ε*_32_ = 0.08; (b) Snapshots of the tip filament illustrating the transition from a stable scroll wave to the onset of its collapse and to turbulent state. (i)*t* = 100, (ii)*t* = 140, (iii)*t* = 160, (iv)*t* = 250.

**Fig 8:**
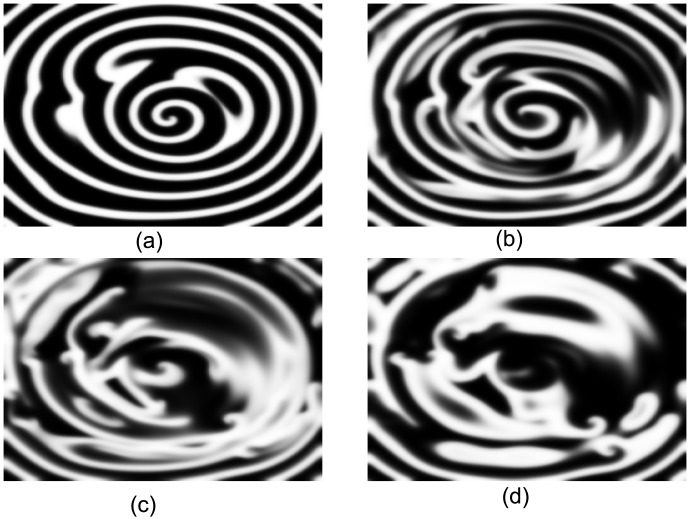
The chaotic motion in four different layers at *t* = 250. (a) the snapshot of the first layer (b) the 10th layer, (c) the 20th layer, (d) the 32th layer. Excitability parameter is *ε* = 0.08.

Spiral waves in 2D medium with *ε* > 0.071 break up because of Doppler instability. When *ε* increases across 0.071 (*ε*_32_ = 0.079), the simulation shows that the spiral wave in the 32nd layer can still be maintained, as plotted in Fig 6. This result is because its meandering behavior, which is characterized by the tip orbit, is moderated for its frequency is increased by the driving of the upper layers. The gradient of excitability is further enhanced with the increase in *ε*_32_. At this point, three dynamical regimes exist along the Z-axis. From Fig 7(a), the spiral waves cannot reach the synchronous state. The twisting of the filament is greatly promoted (Fig 7(bi)) so that the spiral waves in the lower layer cannot be driven any more. Consequently, the scroll wave breaks up. The maximum degree of twisting is in the middle layers (Fig 7(bii)); hence, the break up starts to occur just right here (Fig 7(biii)). Then, a semi-turbulent state is formed, which has been observed and analyzed in recent experiments [18]. This semi-turbulent state can be observed by comparing the patterns in Fig 8 plotted from different layers. In Fig 8(d), the turbulent state in the 32nd layer is presented. Even if the spiral waves in the lower layers break up, the spiral waves in the top layers are still maintained (Fig 8(a)). An interesting phenomenon is that the filament in the top layers, where the spiral waves are retained, is a straight line, as illustrated in Fig 7(biv). This finding indicates that the twist angle in the top layers decreases when break up occurs. Insulating layers seem to exist, which decrease the load and allow the spiral waves to be driven easily in the upper layers.

## Conclusion

We have studied the effects of wave propagation on the twisting of 3D scroll wave filaments in an excitable medium. In our simulation, we change the gradient of parameter *ε* until the system transitions to a turbulent state. Three regimes of Δ*ε* can be distinguished. When the gradient is small, the system reaches a rigidly rotating state with the same modulated period *T*. In this case, the maximum velocity appears in the middle layer where the degree of filament twist is the largest. As the gradient increases, the whole system rapidly achieves meandering synchronization. The top layer with a large excitability is the first to achieve the meandering state. Then, the other rigid spirals in the lower layers are gradually driven to a meandering synchronous state with outward flower-like trajectories from top to bottom. Filament twisting may prevent the breakup of spiral waves in the bottom layers with a low excitability, at which a spiral breaks in a 2D medium. When the *ε* gradient is large enough, the twisting filament is promoted to complicate driving the spirals in the lower layers. Eventually, it breaks up to where the maximum degree of twisting is located. Then, a semi-turbulent state is formed, as illustrated by the maintenance of the linear filament in the upper layers. We believe that our findings predict interesting consequences for the dynamics of scroll waves in heterogeneous media.
